# Co-Precipitation Synthesis of Co_3_[Fe(CN)_6_]_2_·10H_2_O@rGO Anode Electrode for Lithium-Ion Batteries

**DOI:** 10.3390/ma15134705

**Published:** 2022-07-05

**Authors:** Daming Sun, Xiaojie Wang, Meizhen Qu

**Affiliations:** 1 Chengdu Institute of Organic Chemistry, Chinese Academy of Sciences (CAS), No. 9, 4th Section of South Renmin Road, Chengdu 610041, China; mzhqu@cioc.ac.cn; 2 School of Chemistry and Chemical Engineering, Lanzhou Jiaotong University, Lanzhou 730070, China; 12211639@stu.lzjtu.edu.cn

**Keywords:** Prussian blue analog, anode material, rechargeable lithium-ion battery

## Abstract

Rechargeable lithium-ion batteries (LIBs) are known to be practical and cost-effective devices for storing electric energy. LIBs have a low energy density, which calls for the development of new anode materials. The Prussian blue analog (PBA) is identified as being a candidate electrode material due to its facile synthesis, open framework structures, high specific surface areas, tunable composition, designable topologies and rich redox couples. However, its poor electrical conductivity and mechanical properties are the main factors limiting its use. The present study loaded PBA (Co_3_[Fe(CN)_6_]·10H_2_O) on graphene oxide (Co-Fe-PBA@rGO) and then conducted calcination at 300 °C under the protection of nitrogen, which reduced the crystal water and provided more ion diffusion pathways. As a result, Co-Fe-PBA@rGO showed excellent performance when utilized as an anode in LIBs, and its specific capacities were 546.3 and 333.2 mAh g^−1^ at 0.1 and 1.0 A g^−1^, respectively. In addition, the electrode also showed excellent performance in the long-term cycle, and its capacity reached up to 909.7 mAh g^−1^ at 0.1 A g^−1^ following 100 cycles.

## 1. Introduction

In the last 20 years, rechargeable lithium-ion batteries (LIBs) have been extensively utilized in the field of electric vehicles and portable energy storage devices [1]. Nonetheless, economical LIBs that have an extended cycle life as well as a high capacity are urgently needed for many power-hungry mobile electronics and light-duty vehicles [2]. However, the current anode materials, such as graphite, Li_4_Ti_5_O_12_, titania, metal sulfides and silicon, have difficulty meeting the above conditions due to their low specific capacity and poor cycle performance [3,4,5]. Recent studies have focused on finding a new anode material that meets the demand for LIBs.

The Prussian blue analog (PBA) has been proven to be a safe and reliable electrode material due to its low costs, facile synthesis, high specific surface areas, tunable composition, three-dimensional open frameworks, low volume expansion ratio and high theoretical specific capacity (SC) [6,7]. PBA has been widely used in alkali ions [8,9,10], multi-valent ions [11,12,13,14] and microbial batteries [15]. However, PBA suffers from a low capacity and poor electrical conductivity, mechanical properties and cycling stability. Mn-Fe-PBA shows a low specific capacity (372.8 mAh g^−1^) together with decreased cycling performance (with a capacity retention rate (CRR) of 35% following altogether 100 cycles) [8]. The most effective ways to solve this issue are structural optimization and surface medication, especially in combination with highly conductive nanomaterials [16]. Removal of coordination water or zeolite water in the PBA structure and element doping can enhance the structural stability, thus promoting the cycling performance of PBA electrodes. Zn- doped Mn-Fe-PBA with a truncated octahedral structure exhibits a high (519.3 mAh g^−1^) SC at 0.1 A g^−1^ [8].

Moreover, PBA can also be used as a precursor to make other electrode materials including metal oxide/sulfides [17,18], and metal phosphide [19], which have a high specific capacity. The FeMnO_3_ derived from Mn-Fe-PBA delivers a charge capacity as high as 1418.4 mAh g^−1^ [20]. Fe_3_C@N-GE-CNTs obtained from PBA have achieved a high reversible capacity of 1221 mAh g^−1^ when used in Li-S batteries.

Graphene represents one of the two-dimensional (2D) carbon materials, which can be applied in improving electrode mechanical properties along with electroconductivity [21,22,23]. Silicon embedded in sphere graphene and carbon nanotubes exhibits high electrical conductivity, superior mechanical properties (withstanding a pressure of 100 MPa) and outstanding cycling performance (0.014% capacity fading rate at 2 A g^−1^ following 700 cycles) [24].

This study processed in situ fabricated Co_3_[Fe(CN)_6_]_2_·10H_2_O@rGO (Co-Fe-PBA@rGO) for a 2 h period at 300 °C under a N_2_ atmosphere. During the treatment, a portion of the crystal water of Co-Fe-PBA@rGO was removed to make its internal structure more complete, thus providing sufficient ion diffusion paths. Co-Fe-PBA@rGO achieved a capacity as high as 909.7 mAh g^−1^ when utilized as an LIB anode material, demonstrating its application prospect in LIBs.

## 2. Materials and Methods

### 2.1. Synthesis of Co_3_[Fe(CN)_6_]_2_·10H_2_O

First, 15.0 g of decahydrate sodium ferrocyanide (Na_4_[Fe(CN)_6_]·10H_2_O) and 11.6 g of cobalt acetate tetrahydrate (C_4_H_6_CoO_4_·4H_2_O) were dissolved into distilled water (200 mL), and then the mixture was stirred vigorously in a 50 °C water bath. After 10 min, the Na_4_[Fe(CN)_6_]·10H_2_O solution was injected into the C_4_H_6_CoO_4_·4H_2_O solution at a 5 mL min^−1^ flow rate using a peristaltic pump. After the reaction, the solution was left to stand for 12 h, washed with distilled water, centrifuged, freeze-dried for 48 h and then named Co-Fe-PBA. Later, Co-Fe-PBA was processed through a 2 h calcination at 300 °C under a N_2_ atmosphere at a 5 °C min^−1^ heating rate to obtain Co-Fe-PBA-300.

### 2.2. Preparation of Graphene Oxide (GO)

We utilized the Hummers approach after modification for preparing GO [25,26,27,28,29,30]. Details about the production process can be found in the Supporting Information.

### 2.3. Preparation of Co-Fe-PBA@rGO

After placing 15 mL of the above GO suspension in a three-necked flask, the suspension was diluted with 300 mL of deionized water, followed by the immediate addition of 2.06 g sodium hydroxide (NaOH) for adjusting the suspension pH to about 3. Then, 2.63 g C_4_H_6_O_4_·4H_2_O was added with vigorous stirring in a 50 °C water bath. Next, the Na_4_[Fe(CN)_6_]·10H_2_O solution, which was prepared by dissolving Na_4_[Fe(CN)_6_]·10H_2_O (3.4 g) into distilled water (150 mL), was added to the above solution. The freeze-dried samples were handled the same as the Co-Fe-PB-300.

### 2.4. Electrochemical Measurements

For the electrode sheet active material, its areal density was decided to be 0.55 mg cm^−2^. See the Supporting Information for details.

### 2.5. Material Characterization

The characterization instruments and parameters are provided in the Supporting Information.

## 3. Results

**Figure 1:**
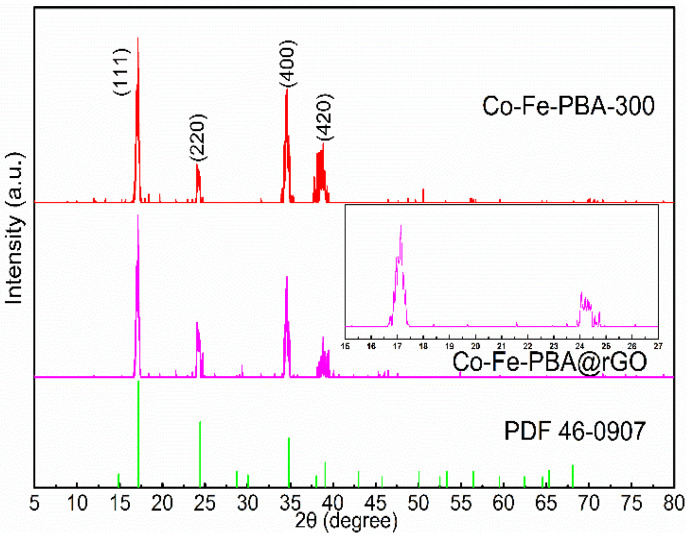
XRD patterns of Co-Fe-PBA-300 and Co-Fe-PBA@rGO.

Figure 1 shows XRD patterns for Co-Fe-PBA-300 together with Co-Fe-PBA@rGO. Their spectra contain characteristic peaks at 14.889°, 24.429°, 34.820° and 39.087°, associated with planes (111), (220), (400) and (420), respectively. The characteristic peaks are completely consistent with the standard card of PDF 46-0907 (the chemical formula is Co_3_[Fe(CN)_6_]_2_·10H_2_O), belonging to the F-43 m (216) space group. The intensity of the Co-Fe-PBA-300 diffraction peaks increased at 24.429° and decreased slightly at 39.087°, which was due to the GO layer limiting the growth direction and rate of Co-Fe-PBA, which can also prevent the agglomeration of the Co-Fe-PBA nanoparticles [31].

**Figure 2:**
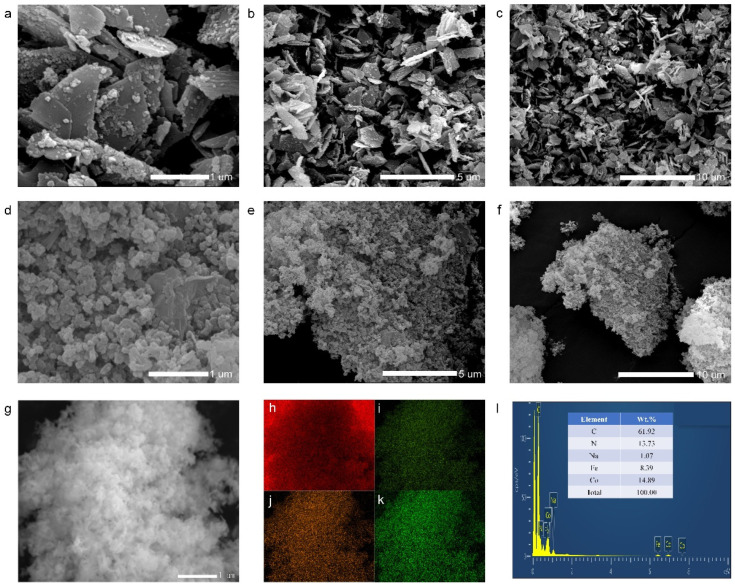
SEM images of (**a**–**c**) Co-Fe-PBA-300 and (**d**–**g**) Co-Fe-PBA@rGO; (**h**,**l**) EDS mapping of Co-Fe-PBA@rGO: (**h**) C, (**i**) Co, (**j**) Fe and (**k**) N and (**l**) EDS spectrum.

Figure 2 and Figure S1 present field-emission scanning electron microscopy (FESEM) images for Co-Fe-PBA-300 as well as Co-Fe-PBA@rGO. In terms of the blank Co-Fe-PBA-300 (Figure 2a–c), there are many cubic particles anchored on the flake-like sheets. When Co-Fe-PBA grows in situ in the GO suspension, Co-Fe-PBA attaches to GO in an irregular cubic shape without a flake-like appearance, which is because the GO layer limits the growth direction and rate of Co-Fe-PBA. This is consistent with the XRD. The EDS mapping shown in Figure 2g–l indicates a homogeneous distribution of all elements.

**Figure 3:**
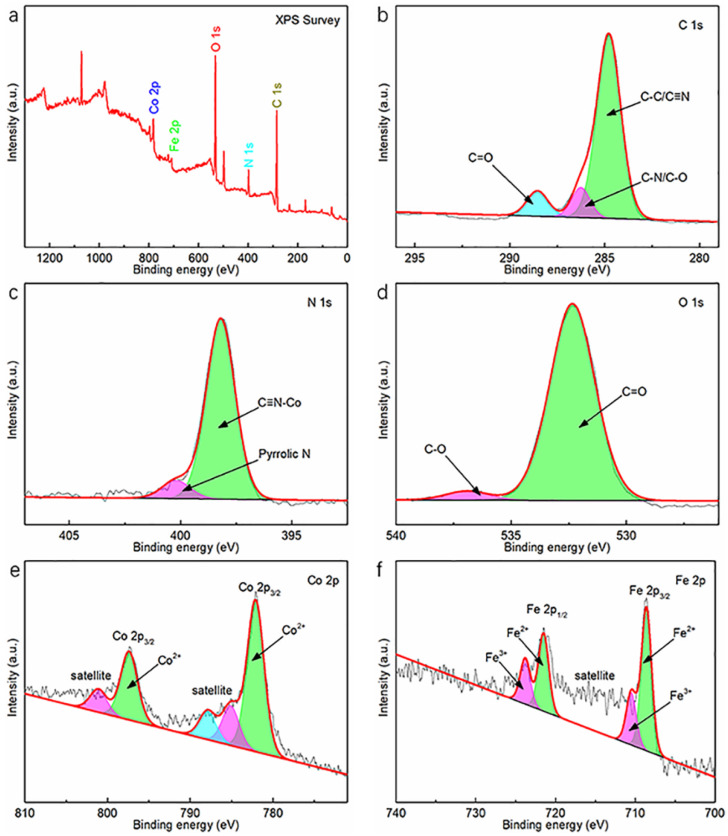
(**a**) Survey XPS spectrum of the fresh Co-Fe-PBA@rGO composite; high-resolution XPS spectra of (**b**) C 1s peaks, (**c**) N 1s peaks, (**d**) O 1s peaks, (**e**) Co 2p peaks and (**f**) Fe 2p peaks.

Figure 3 illustrates an X-ray photoelectron spectroscopy (XPS) image for the Co-Fe-PBA@rGO composite. The image was utilized to determine the elemental valence and composition. Figure 3a displays the survey spectrum for Co-Fe-PBA@rGO. The binding energies of C 1s, N 1s, O 1s, Co 2p and Fe 2p were located at 284.8, 399, 532, 710 and 779 eV, respectively. The C 1s can be fitted to three peaks detected at 284.8, 286.3 and 288.5 eV, which were related to C-C/C≡N, C-N/C-O and C=O, respectively (Figure 3b). The presence of abundant C-C/C≡N indicates that the structures of the sp^2^ hybrid C-C, as well as the Co-Fe-PBA within the Co-Fe-PBA@rGO, were not destroyed after treatment at 300 °C under a N_2_ atmosphere. The appearance of C-C suggests the destruction of rGO within a composite during the calcination process. In addition, the small amounts of C-C and C=O demonstrate that the GO in the Co-Fe-PBA@rGO composite was partially reduced. The C-N at 288.5 eV indicates that part of the GO was nitrided during calcination in the N_2_ atmosphere. 

The process is easy to achieve because the radius of the N atom is close to the C atom. The introduced N replaces part of the C on the 2D plane of the GO skeleton. Nitrogen doping can reduce the oxygen-containing functional group quantity on the Co-Fe-PBA@rGO layer surface, while skeleton doping can form more defects, which facilitates the electrical and mechanical performances of Co-Fe-PBA@rGO composites. The same conclusion can be drawn from the fitted peak of N 1s (Figure 3c). The 400.4 eV peak indicates that the sp^2^ hybrid N is connected to the sp^2^ hybrid C in the rGO skeleton to form pyrrole nitrogen. That is, N provides two electrons to pair with the C in the GO skeleton when calcined in N_2_, and the remaining electron on N contributes to improving the electrical conductivity of the whole Co-Fe-PBA@rGO composite. 

Apart from that, the peak at 398.2 eV is mainly derived from the sp hybrid N, attributed to C≡N-C, indicating that the N in the Prussian blue structure accounts for a large proportion of the total and that the high-spin Co^2+^ is connected to the N in Co-Fe-PBA@rGO. After calcination at 300 °C in an inert atmosphere, part of the bound water and all the free water adsorbed in the crystal structure of the Co-Fe-PBA@rGO composite can be removed. Thus, the fitted peaks of O 1s (Figure 3d) only show C=O at 532.3 eV, whereas C-O is shown at 536.9 eV. Typically, the C-O peak area and intensity are significantly smaller than those of the C=O peak. However, the intensity and area of C-N/C-O are equal to those of the C=O in Figure 3b, which indicates a mass of C-N in C-N/C-O, strongly proving that GO does introduce N during calcination. As observed from the high-resolution XPS spectrum for Co 2p (Figure 3e), two peaks at 782.1 eV (Co 2p_3/2_) and 797.4 eV (Co 2p_1/2_) were associated with Co^2+^, while the other three peaks at 785.1 eV (Co 2p_3/2_), 787.9 eV (Co 2p_3/2_) and 801.1 eV (Co 2p_1/2_) were the satellite peaks of the former two. The reason for this is mainly the formation of vacancy in the inner layer of Co and the sudden change in its central potential, which leads to the transition of the outer electrons to higher energy levels and the emergence of vibration peaks. It can be seen that Co exists in the bivalent form in the Co-Fe-PBA@rGO composite, which well conforms to Co_3_[Fe(CN)_6_]_2_·10H_2_O’s chemical formula. Figure 3f reveals the fitted results of Fe 2p. The two peaks belonging to Fe^2+^ were located at 708.6 eV (Fe 2p_3/2_) and 721.5 eV (Fe 2p_1/2_), whereas the 710.5 eV (Fe 2p_3/2_) and 723.8 eV (Fe 2p_1/2_) peaks were associated with Fe^3+^ [32]. A relatively large satellite peak exists between Fe 2p_3/2_ and Fe 2p_1/2_. In addition, the peak belongs to the region of electron transition to the unbound continuous region and becomes the vibration peak of the free electron. The above results show that Fe^2+^ and Fe^3+^ co-exist in the Co-Fe-PBA@rGO composite [33,34,35]. 

**Figure 4:**
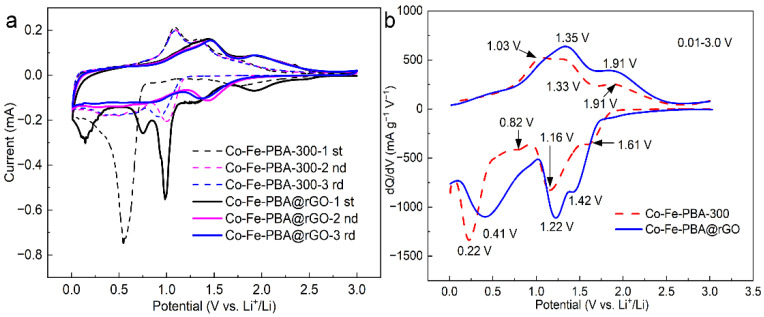
(**a**) CV curves for Co-Fe-PBA-300 and Co-Fe-PBA@rGO for the first 3 cycles at a 0.1 mV s^−1^ scan rate and 0.01–3.0 V voltage vs. Li^+^/Li; (**b**) dQ/dV curves (Q, capacity; V, voltage) vs. the initial discharge–charge profiles for fabricated Co-Fe-PBA-300 together with Co-Fe-PBA@rGO at a 0.1 A g^−1^ current density.

This work utilized cyclic voltammetry (CV) to determine redox reactions occurring on the electrode during charging and discharging. The curves for Co-Fe-PBA-300 and Co-Fe-PBA@rGO at a 0.1 mV s^−1^ scan rate and 0.01–3.0 V voltage vs. Li^+^/Li are illustrated in Figure 4a. For Co-Fe-PBA-300, three reduction peaks were observed at 1.73, 0.94 and 0.53 V in the initial discharge cycle. Meanwhile, the 1.73 V peak was caused by the reduction of Co^3+^ to Co^2+^ and disappeared during the following cycle, which suggests an irreversible reaction. The peak at 0.94 V indicates that the Co^2+^ generated in the previous step and contained in the Co-Fe-PBA-300 was further reduced. During the second discharge cycle, this peak not only increased in intensity but also split into two peaks at the 1.0 (high) and 0.69 (low) V voltages. In the third discharge cycle, the above two reduction peaks migrated to the 0.92 and 0.66 V low voltages, respectively, which shows that the redox reaction at this stage was completely reversible and stable, further proving that the Co ions were gradually reduced. The strong peak at 0.53 V included not only the decomposition of lithium salts and solvents in the electrolyte to form SEI films, but also the reduction process of Fe^3+^ [36]. In the second cycle, this peak decreased significantly in intensity and split into two 0.52 and 0.36 V reduction peaks. These two peaks basically coincided during the reduction in the third cycle, indicating that Fe^3+^ can be reduced completely and that the loss of metal lithium caused by irreversible side reactions and the formation of SEI mainly occurred during the initial reduction reaction. There were three oxidation peaks situated at 1.09, 1.34 and 1.72 V, basically coinciding with the first cycle, which indicates that the above process achieved excellent cycle stability and reversibility.

The CV curve of the first cycle discharge of Co-Fe-PBA@rGO showed five reduction peaks at 1.92, 1.32, 0.98, 0.75 and 0.15 V. The introduction of nitrogen atoms into rGO can not only increase the band gap of rGO [37,38] but also change the electronic structure around graphene [39,40,41], thus increasing the carrier density of graphene. Graphene’s electrical conductivity and mechanical stability can be improved [42], and more active sites can be provided for the adsorption of metal particles on the surface [43]. Therefore, the three reduction peaks of Co-Fe-PBA@rGO at 1.73, 0.94 and 0.53 V in the Co-Fe-PBA@rGO samples migrated to 1.92, 1.32 and 0.98 V, respectively. rGO can effectively prevent the contact of the Co-Fe-PBA nanoparticles with the electrolyte. The intensity and area of the peak of the Co-Fe-PBA@rGO composite at 0.98 V were small due to the lower number of side reactions and electrolyte decomposition. In addition, the 0.75 V peak indicates that the oxygen-containing functional groups on the rGO layers consumed a lot of Li^+^, along with partial electrolyte decomposition, in order to form the SEI film [29]. In addition, the peak at 0.15 V indicates that some Li^+^ was embedded in the rGO layers. 

In the oxidation process, all the oxidation peaks migrated to high potentials at 1.13, 1.46 and 1.91 V, respectively. The second oxidation peak of Co-Fe-PBA@rGO changed very little in intensity and area compared to that of Co-Fe-PBA-300. However, the first oxidation peak became lower and wider, almost overlapping with the second one, while the third oxidation peak increased significantly. From the second cycle, the oxidation peak was covered completely, indicating that the electrode had good reversibility and cycle stability. This phenomenon occurs mainly because the rGO layer can prevent the agglomeration and fragmentation of Co-Fe-PBA nanoparticles and reduce the corrosion rate and degree of Co-Fe-PBA nanoparticles by the electrolyte. Moreover, Co-Fe-PBA nanoparticles can avoid rGO layer accumulation, and the synergistic effect of Co-Fe-PBA and rGO enables the composites to possess good mechanical stability [44]. The dramatic increase in the oxidation/reduction potential and intensity was related to the increase in surface area [31].

Figure 4 presents dQ/dV curves as a function of the initial discharge–charge profiles for Co-Fe-PBA-300 and the Co-Fe-PBA@rGO composite at a 0.1 A g^−1^ current density. During the reduction of Co-Fe-PBA-300, there were four reduction peaks at 1.61, 1.16, 0.82 and 0.22 V, while there were only three reduction peaks of the Co-Fe-PBA@rGO composite at 1.42, 1.22 and 0.41 V. Since the SEI film was formed from 0.8 V on rGO, the peaks at 0.82 and 0.22 V were combined. Similarly, for the Co-Fe-PBA@rGO composite, the peaks in the oxidation process detected at 1.03 and 1.33 V merged and migrated to high potentials.

**Figure 5:**
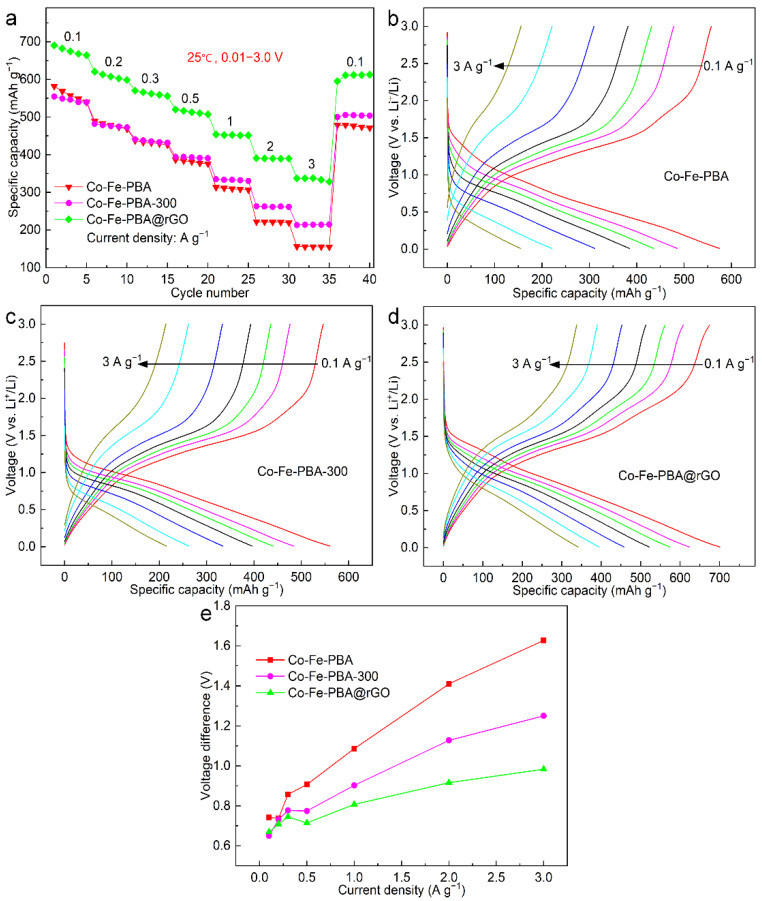
(**a**) Rate capabilities of Co-Fe-PBA and Co-Fe-PBA-300, together with Co-Fe-PBA@rGO, under 0.1–3.0 A g^−1^ current densities; (**b**–**d**) respective discharge–charge curves under diverse current densities over 3 cycles; (**e**) the relation between charge–discharge voltage difference and current density.

Figure 5a shows the rate performance of Co-Fe-PBA, Co-Fe-PBA-300 and Co-Fe-PBA@rGO under a 0.01–3.0 V voltage and 0.1/0.2/0.3/0.5/1.0/2.0/3.0 A g^−1^ current densities. Co-Fe-PBA’s specific charge capacity at the above current densities was 557.4, 478.2, 431.7, 381.5, 309.6, 221.3 and 155.5 mAh g^−1^ (all of which were taken from the values in the third cycle at each charge–discharge current density), corresponding to CRRs of 100%, 85.8%, 77.4%, 68.4%, 55.5%, 39.7% and 27.9%, respectively. The specific charge capacities of Co-Fe-PBA-300 at the above current densities were 546.3, 476.1, 435.6, 393.2, 333.2, 261.5 and 214.1 mAh g^−1^, associated with the 100%, 87.1%, 79.7%, 72.0%, 61.0%, 47.9% and 39.2% CRRs, respectively. Clearly, at the <0.5 A g^−1^ discharge–charge current density, there existed almost no obvious difference in the charge specific capacity (CSC) between Co-Fe-PBA and Co-Fe-PBA-300. However, Co-Fe-PBA-300 showed a higher specific capacity with the increasing charge–discharge current density. At higher charge–discharge current densities, Co-Fe-PBA-300 had increased capacity retention compared with Co-Fe-PBA. The reason lies in that the structure of PBA prepared directly by traditional co-precipitation methods is mostly incomplete and unstable due to the rapid precipitation rate. The vacancy and crystal water in the crystal structure significantly affect the electrochemical properties [45,46]. In this work, the crystal water in Co-Fe-PBA was removed after calcination at 300 °C in a N_2_ atmosphere, providing sufficient diffusion paths for lithium ions [47]. Therefore, Co-Fe-PBA-300 showed a superior rate performance and CRR compared with Co-Fe-PBA, particularly at the increased charge–discharge rates.

The specific capacities of the Co-Fe-PBA@rGO composite were 674.3, 607.8, 561.9, 513.0, 452.6, 389.9 and 337.9 mAh g^−1^. The Co-Fe-PBA@rGO composite had a higher CRR and SC than those of Co-Fe-PBA and Co-Fe-PBA-300. This result was due to the C-N bond formed by the pyrrole nitrogen connected to the rGO five-membered ring, which provided more active centers to promote the reaction between the carbon skeletons and lithium ions.

Figure 5b–d display charge–discharge curves for the third cycle corresponding to the different charge–discharge current densities in Figure 5a. For Co-Fe-PBA, the discharge curve presents a steep diagonal line, and the platform voltage decreased from 1.0 V at 0.1 A g^−1^ to about 0.5 V at 3 A g^−1^. Similarly, the charge platform at 1.0 V almost disappeared at the 3 A g^−1^ current density, which indicates the strong influence of the charge–discharge current density on the redox reaction at 1.0 V. This phenomenon occurs mainly because of kinetic reasons, including high SEI impedance and a low lithium-ion diffusion coefficient [48]. However, the shape and specific capacity of the charging curve within 2.2–3.0 V did not change significantly, suggesting the good reversibility and cycle stability of the redox reaction in this voltage range. 

The length and voltage of the charge–discharge platform of Co-Fe-PBA-300 increased slightly. The Co-Fe-PBA grown on the rGO layer still maintained a charge platform of about 1.0 V under the high current densities, probably because of the excellent electronic conductivity of rGO. After calcination in the N_2_ atmosphere, the graphene skeleton doped with the pyrrole nitrogen further improved the electrochemical performance. As a result, the electronic conductivity of Co-Fe-PBA@rGO was improved remarkably. As shown in the above three figures, as the current density increased, the discharge–charge platforms of the Co-Fe-PBA, Co-Fe-PBA-300 and Co-Fe-PBA@rGO composites migrated to low and high voltages, respectively, resulting in a certain degree of polarization. 

In addition, a phenomenon can be seen from the relation between the charge–discharge voltage difference and the current density in Figure 5e. The voltage difference was small among the three at the <0.3 A g^−1^ current density. However, the polarization became increasingly significant at the >0.5 A g^−1^ current density, and the polarization of the Co-Fe-PBA@rGO composite was less significant than that of the other two. This result is mainly because the pyrrolidine nitrogen (C=N-C) introduced to the rGO forms certain carbon defects around it [49], which provides many effective paths for electron and lithium ion transport and improves the transmission efficiency. Hence, it has a high rate performance and SC [50,51].

**Figure 6:**
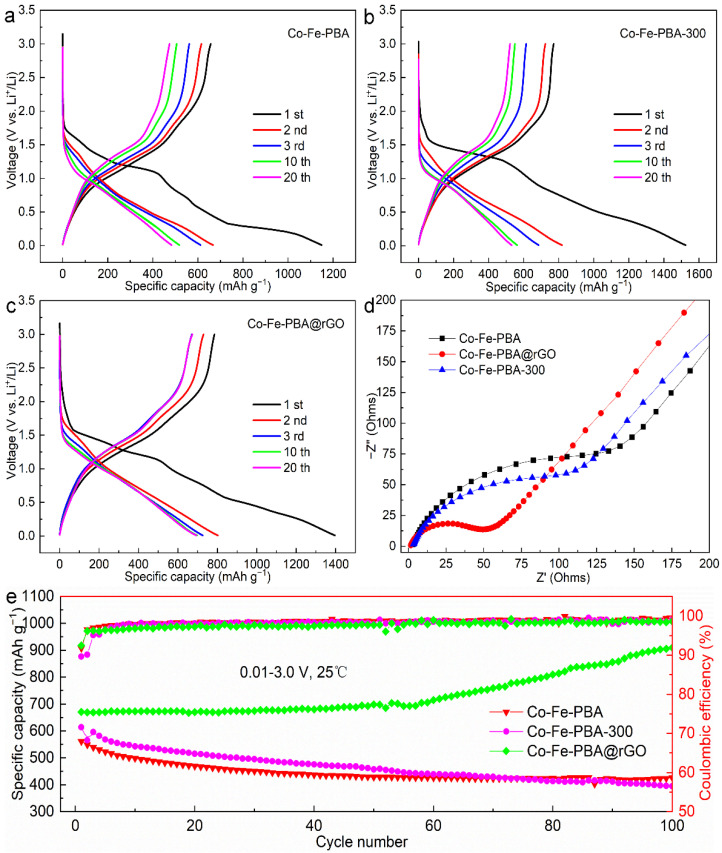
Electrochemical performance of Co-Fe-PBA, Co-Fe-PBA-300 and Co-Fe-PBA@rGO composite anode materials: (**a**–**c**) galvanostatic discharge–charge voltage profiles; (**d**) Nyquist plots; (**e**) long cycling life at 0.1 A g^−1^ under a 0.01–3.0 V voltage.

Figure 6 shows the charge–discharge curves, cycle performance and EIS impedance spectra of the Co-Fe-PBA, Co-Fe-PBA-300 and Co-Fe-PBA@rGO composites at the 0.1 A g^−1^ current density and 0.01–3.0 V voltage. According to Figure 6a, four platforms appeared at 1.25, 0.8, 0.5 and 0.25 V during the first discharge cycle. The LiPF_6_ in the electrolyte irreversibly decomposed to form SEI at 0.8 V. The other three were irreversible reactions from when the lithium ions entered into the Co-Fe-PBA nanoparticles. In the second cycle of discharge, the four platforms almost disappeared into an inclined straight line, indicating that the formation of SEI and most irreversible reactions occurred during the first reduction. The original discharge platform at 1.25 V dropped to about 1.0 V after 10 cycles of activation. After 20 cycles, the discharge curve mostly coincided with the 10th cycle, indicating that the electrode structure reached a stable level. During the first charge, there were three platforms at 1.0–1.5 V, 1.5–2.0 V and 2.0–3.0 V, where the first platform had the largest specific capacity. In the subsequent cycles, the charge platform within 1.0–1.5 V was gradually shortened, and the platform SC decreased by 130 mAh g^−1^ after 20 cycles. Moreover, the charge and discharge specific capacities in the first cycle were 656.6 and 1149.2 mAh g^−1^, respectively, which equaled a 57.1% Coulomb efficiency. 

As shown in Figure 6b, during the first discharge of Co-Fe-PBA-300, the platform at 1.5 V becomes flatter, and the discharge curve from the lower voltage to the cut-off voltage is almost an inclined straight line, which equals a 1523.1 mAh g^−1^ discharge specific capacity (DSC). The next discharge curve is not significantly different from that of Co-Fe-PBA. However, with regard to the discharge curve, the 10th cycle was the same as the 20th cycle, suggesting that the electrode remained stable from the 10th cycle. The specific capacity of the first charge reached 771.0 mAh g^−1^, with a 50.6% initial efficiency. After high-temperature treatment, the first charge efficiency remained lower than that of the blank Co-Fe-PBA, although the first charge capacity increased by 114.4 mAh g^−1^. This is mainly because Co-Fe-PBA-300 lost part of the bound water and generated more new vacancies in the structure to accommodate lithium ions, as the first DSC increased to 373.9 mAh g^−1^. 

The discharge platform of the Co-Fe-PBA@rGO composite (Figure 6c) was close to that of Co-Fe-PBA when the discharge voltage was above 1.0 V and similar to that of Co-Fe-PBA-300 at the <1.0 V discharge voltage. The platform at 0.8 V of the Co-Fe-PBA@rGO composite is more significant, mainly because the oxygen-containing functional groups on the rGO layers consumed a substantial amount of lithium ions irreversibly, inducing a 1396.1 mAh g^−1^ DSC. The specific capacities of the platform at 1.0–1.5 V and 1.5–2.0 V increased significantly in the charge process and showed excellent stability in the subsequent cycles. The first charge capacity reached 785.0 mAh g^−1^, with a corresponding 56.2% initial Coulombic efficiency. Noteworthily, in the third cycle, the charge and discharge curves matched perfectly, and the electrode reached a stable state rapidly. The above experimental results can also be confirmed by the EIS impedance spectra of the uncycled batteries (Figure 6d). Obviously, the bulk-phase impedance and interface impedance of the Co-Fe-PBA@rGO composite were lower than those of Co-Fe-PBA and CoFe-PBA-300. However, the complete opposite is true in terms of the ion diffusion rate.

Figure 6e shows the cycle performances of Co-Fe-PBA and Co-Fe-PBA-300, together with Co-Fe-PBA@rGO, under the 0.1 A g^−1^ current density and 0.01–3.0 V voltage. For Co-Fe-PBA and Co-Fe-PBA-300, their CSC decreased with the increasing cycle numbers. After 100 cycles, the capacity of Co-Fe-PBA decreased from 561.3 to 426.6 mAh g^−1^, whereas that of Co-Fe-PBA-300 decreased from 614.1 to 394.6 mAh g^−1^, yielding 76% and 64.3% CRRs, respectively. However, the cycle performance of Co-Fe-PBA@rGO was completely different from that of Co-Fe-PBA and Co-Fe-PBA-300. During the first 50 cycles, the specific capacity increased slightly from 670.6 mAh g^−1^ to 698.6 mAh g^−1^. However, from the 50th cycle to the 100th cycle, the specific capacity increased sharply to 909.7 mAh g^−1^, showing a good cycle performance. The main reasons for this phenomenon are as follows: Firstly, the electrochemically active film formed gradually during the cycling process can prevent the aggregation and restrain the volume effect of the PBA nanoparticles. Secondly, the progressively increased specific surface area caused by the transformation of large particles to small particles on rGO can provide an abundance of Li^+^ storage centers [52].

## 4. Conclusions

Co_3_[Fe(CN)_6_]_2_·10H_2_O was prepared by the traditional co-precipitation method. It had a high SC but low cycling stability when utilized as an LIB anode material. Its SC further increased because part of the bound water in the crystal was lost after it was calcined in a N_2_ atmosphere at 300 °C. However, there was no significant change in the cycle performance due to the low electroconductivity. The capacity of the Co-Fe-PBA@rGO composite was obviously increased, with a superb cycling stability and rate capability. After 50 cycles of activation, the charge specific capacity increased dramatically to 909.7 mAh g^−1^ when utilized as an LIB anode.

## Data Availability

All the involved data are available upon request.

## Supplementary Materials

The following supporting information can be downloaded at: https://www.mdpi.com/article/10.3390/ma15134705/s1, Figure S1: High-resolution SEM images of Co-Fe-PBA@rGO.
