# Supplementary material for: Co-Precipitation Synthesis of Co3[Fe(CN)6]2·10H2O@rGO Anode Electrode for Lithium-Ion Batteries
Source: Materials (Basel). 2022 Jul 5;15(13):4705. doi: 10.3390/ma15134705 (PMC9267929; doi:10.3390/ma15134705)
Supplement: Supplementary file 1 [file materials-15-04705-s001.zip › materials-1769211-supplementary.pdf]

Supporting Information

Co-Precipitation Synthesis of $Co_3[Fe(CN)_6]_2\cdot 10H_2O$@rGO Anode Electrode for Lithium-Ion Batteries

Daming Sun [1,2,*], Xiaojie Wang [2] and Meizhen Qu [1]

[1] Chengdu Institute of Organic Chemistry, Chinese Academy of Sciences (CAS), No. 9, 4th Section of South Renmin Road, Chengdu 610041, China; mzhqu@cioc.ac.cn
[2] School of Chemistry and Chemical Engineering, Lanzhou Jiaotong University, Lanzhou 730070, China; 12211639@stu.lzjtu.edu.cn.
* Correspondence: sundaming@mail.lzjtu.cn

2.2. Synthesis of graphene oxide (GO)

The graphene oxide was synthesized as the process of modified Hummers method. Specifically, 96 mL of concentrated sulfuric acid (analytical grade, AR, Chengdu Kelong Chemical Co., Ltd., China) was slowly added into the mixture of expanded graphite (4 g, Qingdao Yanhai carbon materials Co., Ltd., China) and $NaNO_3$ (2 g, AR, Chengdu Kelong Chemical Co., Ltd., China) in an ice-water bath for security. Subsequently, 12 g of $KMnO_4$ (AR, Chengdu Kelong Chemical Co., Ltd., China) was added into the mixture little by little. At the same time, the suspension was stirred at constant speed for 24 h. The paste was then heated to 40 °C and maintained for 30 min, after that, 200 mL of deionized water was dropwise added into the paste, followed by an increasing of temperature to 90 °C and kept for 30 min. 666 mL deionized water and some H_2O_2 solution (wt.%, 30%, AR, Chengdu Kelong Chemical Co., Ltd., China) were stirred into above solution until no effervescence was appeared. Lastly, the suspension was treated with ultrasonic dispersion for 30 min and stood overnight at room temperature.

2.4. Electrochemical measurements

The slurry was mixed by active materials, Super P (Imerys Graphite & Carbon, Switzerland) and LA133 (Chengdu Indigo Power Sources Co., Ltd., China) in a weight ratio of 80:10:10. The electrode was dried at 105 °C in vacuum for 12 h. The mass loading level of the electrodes was about 0.55 mg cm^{-2}. 2032 coin cells were assembled by circular electrode with a diameter of 12 mm, polypropylene membrane separator (Celgard 2400, LLC, USA) and Li metal (diameter=16 mm, thickness: 0.6 mm, China Energy Lithium Co., Ltd., China) in an argon-filled glovebox. The electrolyte was composed of 1.0 M $LiPF_6$ and 2 wt.% vinylene carbonate (VC) in ethylene carbonate (EC)/diethylene carbonate (DEC)/dimethyl carbonate (DMC) (volume ratio=1:1:1, Shenzhen Capchem Technology Co., Ltd., China). The galvanostatic discharge/charge tests were performed on Neware CT-4008 battery testing system (Shenzhen Neware Technology Co., Ltd., China). Cyclic voltammetry (CV, 0.01-3.0 V vs. Li^+/Li) studies and EIS data (frequency range: 100 kHz to 0.01 Hz) were recorded on a Autolab M204 (Metrohm, Switzerland) electrochemical workstation.

2.5. Material characterization

The morphology and element composition of the sample were determined by field emission scanning electron microscopy (FESEM) (ZEISS GeminiSEM 500, Germany) and X-ray photoelectron spectroscopy (XPS) (XSAM800, Kratos, UK). The crystal structure was obtained from Powder X-ray diffraction (XRD) (EMPYREAN, PANalytical B. V., Holland), using a Cu Kα radiation at 40 kV, 40 mA. The Raman spectra were carried on laser Raman spectrometer (LabRAM HR, HORIBA, France). The thermal stability of the as-prepared sample was determined by thermogravimetric analysis (TGA) (STA 449 F3 Jupiter®, NETZSCH, Germany) under N_2 flow with a heating rate of 10 °C min^{-1}.

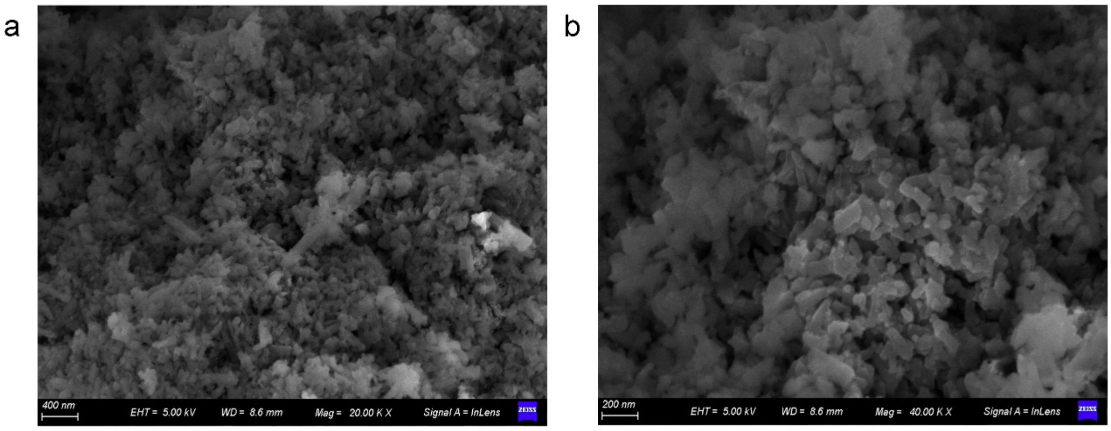

Figure S1. High-resolution SEM images of Co-Fe-PBA@rGO: (**a**) 20.00 *KX* and (**b**) 40.00 *KX*.
